# Exploring the effect of novel six moments on hand hygiene compliance among hospital cleaning staff members: a quasi-experimental study

**DOI:** 10.1017/S0950268823000602

**Published:** 2023-04-28

**Authors:** Wenbin He, Xiaoyan Chen, Xiaolin Cheng, Yan Li, Bilong Feng, Ying Wang

**Affiliations:** 1 Department of Nursing, Zhongnan Hospital of Wuhan University, Wuhan, China; 2 Office of Healthcare-Associated Infection Management of Wuhan University, Wuhan, China; 3 Hubei Engineering Center for Infectious Disease Prevention, Control and Treatment, Wuhan, China

**Keywords:** Compliance, hand hygiene, healthcare, infection, intervention study

## Abstract

My 5 moments (M5M) was used less frequently among cleaning staff members, suggesting that a poor compliance score in this group may not indicate deficient handwashing. This quasi-experimental study compared hand hygiene compliance (HHC), hand hygiene (HH) moments, and HH time distribution in the control group (no HH intervention; *n* = 21), case group 1 (normal M5M intervention; *n* = 26), case group 2 (extensive novel six moments (NSM) training; *n* = 24), and case group 3 (refined NSM training; *n* = 18). The intervention’s effect was evaluated after 3 months. The HHC gap among the four groups gradually increased in the second intervention month (control group, 31.43%; case group 1, 38.74%; case group 2, 40.19%; case group 3, 52.21%; *p* < 0.05). After the intervention period, the HHC of case groups 2 and 3 improved significantly from the baseline (23.85% vs. 59.22%, 27.41% vs. 83.62%, respectively; *p* < 0.05). ‘After transferring medical waste from the site’ had the highest HHC in case group 3, 90.72% (95% confidence interval, 0.1926–0.3967). HH peak hours were from 6 AM to 9 AM and 2 PM to 3 PM. The study showed that the implementation of an NSM practice can serve as an HHC monitoring indicator and direct relevant training interventions to improve HH among hospital cleaning staff.

## Introduction

Hand hygiene (HH) is one of the most effective prevention and control measures for healthcare-associated infections (HAIs). Pittet [1] showed that after hand hygiene compliance (HHC) increased (47.6% to 66.2%), the incidence of HAIs decreased from 16.9% to 9.9%. During the COVID-19 pandemic, HH proved to be one of the most effective prevention and control measures among medical staff members and the public worldwide [2, 3]. Nevertheless, according to a recent study [4], the compliance rate in developed countries was twice that recorded (40% vs. 20%) in developing countries.

The World Health Organization (WHO) Issued guidelines for the implementation and practice of HH in medical institutions in 2009 [5, 6], which proposed the concept of ‘my 5 moments’ (M5M) for HH for medical personnel; these are i) before touching a patient, ii) before cleaning/aseptic procedures, iii) after risk of exposure to body fluids, iv) after touching a patient, and v) after touching a patient’s surroundings [7, 8]. The proposal proved to play a critical role in improving the HH compliance of staff members in medical institutions. Other measures such as the adoption of a combination of the quality control circle and plan–do–check–act were also found to be effective [9]. Likewise, some studies have sought to maintain adherence via reminder mechanisms such as posters to improve HH [10, 11], and an Internet-based tool to monitor and test the HH compliance of nursing and medical staff members also appears to be effective [12]. Other studies have explored the training and promotion of HH practice among clinical staff [13, 14]. Nevertheless, a comprehensive review of the recent literature has highlighted a paucity of studies on HHC among cleaning staff in medical institutions [15]. Such staff play a crucial role in hospitals as they work in all clinical and administrative areas to clean and remove medical and other waste as well as disinfection of floors and furniture surfaces. Consequently, their hands pose a significant risk of transmitting cross-infection microbes in the wider work environment.

Although the majority (63%) of 57 studies cited by Clancy et al. [15] followed the WHO multimodal framework, and most documented HH opportunities at each of the M5M points, few have recorded the HH technique used. We surmised that the practice of M5M was used less frequently among cleaning staff members, which suggests that a poor compliance score may not necessarily indicate deficient handwashing practice, or that a good score might also not reflect adequacy. For example, three specific moments relating to direct patient contact (i.e., i, ii, and iv as outlined earlier) are rarely noted among cleaning staff and, hence, are not monitored to allow an objective evaluation of an intervention effect. We, therefore, developed a risk identification and cluster analysis method to establish a ‘novel six moments’ (NSM) more applicable to cleaning which included (1) before cleaning and disinfection, (2) after preparation of tools, (3) after cleaning and disinfection, (4) after doffing personal protective equipment, (5) after transferring medical waste from the site, and (6) after environmental sorting of waste [16]. However, the efficacy of the N6M strategy for monitoring the effect of HHC intervention between M5M and N6M practices among hospital cleaning staff remains to be determined. This study, therefore, aimed to explore the effect of N6M on HHC among hospital cleaners to address a potentially significant knowledge gap in HAI control from the perspectives of training and compliance monitoring of this staff group.

## Materials and methods

### Study setting

This quasi-experimental study was conducted at Zhongnan Hospital of Wuhan University in Wuhan, a national tertiary hospital with 3,300 beds and 141 cleaning staff members. Ethics approval was obtained from the Ethics Review Committee of the University (2022037K). All participants provided written informed consent.

### Study population

**Figure 1.**
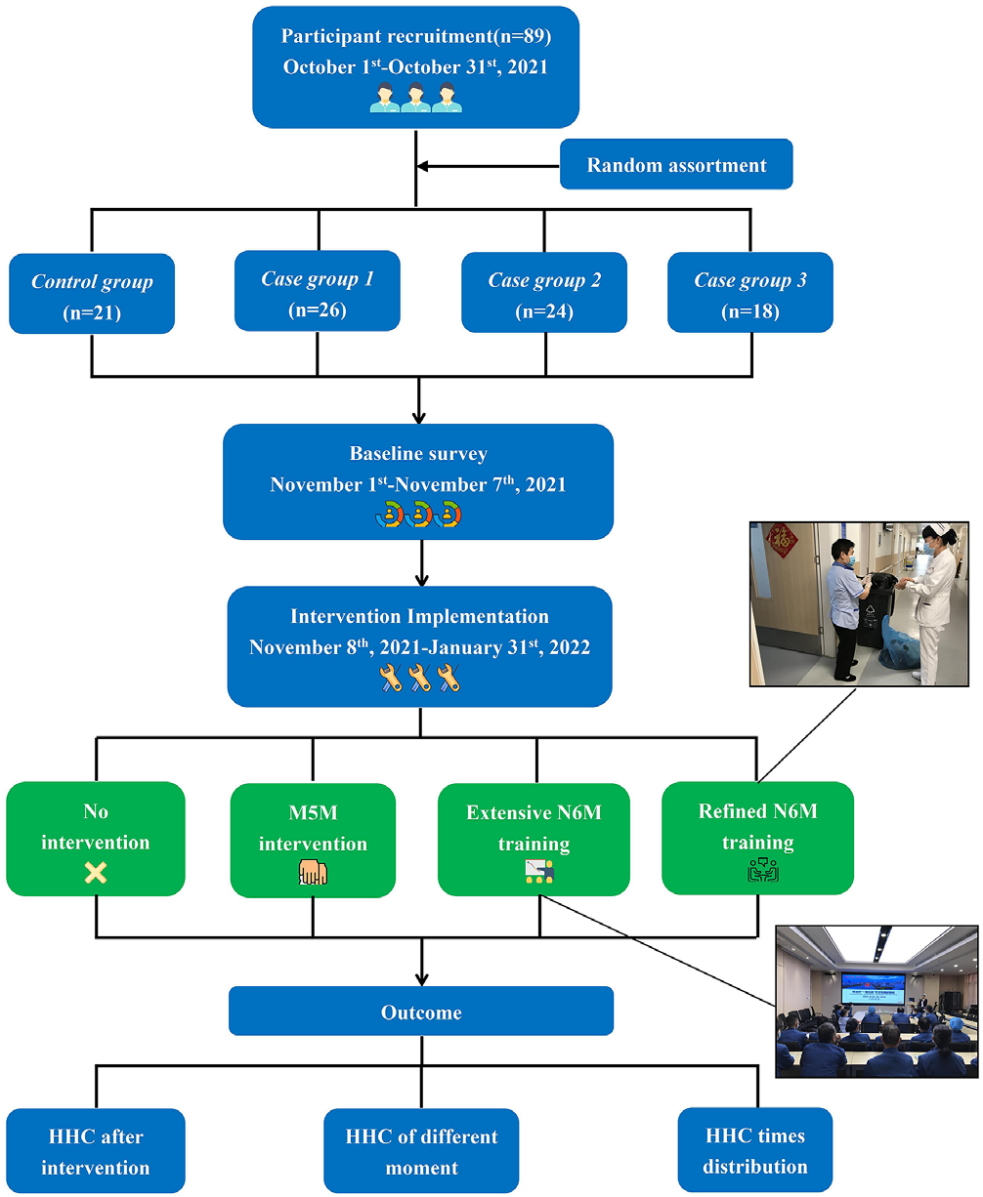
Flowchart.

From November 2021 to January 2022, 89 hospital cleaning staff were selected as participants (Figure 1) if they i) had been employed for three or more months, ii) had no participation in standard training, iii) had a level of cultural knowledge, iv) were under 75 years of age, and v) have no damage to hands. Excluded staff included those leaving employment during the study, did not wish to participate, or were allergic to hand sanitizer. Staff were mainly responsible for cleaning and disinfection of the working area of the inpatient department, including floors, workstations, ward walls, and bed units, and the classification, packaging, and sorting of medical waste. The project team comprised three head nurses, two infection/prevention personnel, and their HH supervisors, who were responsible for collecting the data, communicating with consenting cleaning staff in detail regarding the project, and conducting the study.

### Study design

The participants were randomly divided into four groups: control group, case group 1, case group 2, and case group 3 according to four inpatient buildings. A baseline survey for HHC was conducted among all participants in November 2021, and an intervention programme for N6M was undertaken in two case groups from then until January 2022. Due to the four different locations, there was no staff overlap, or intergroup communication when working, and no possibility of cross-contamination between the locations. Except for the control group, the other three groups remained consistent in intervention duration and frequency, and trainers. The detailed intervention methods for the four groups were as follows:

Control group: 21 participants who did not receive HH training.

Case group 1: 26 cleaning staff members trained in the six-step washing technique, who also underwent regular M5M training as recommended by the WHO [5]. At the beginning of each of the three months, the full-time hospital infection trainer organised 30-minute training sessions for the cleaning staff, which addressed HH concepts, methods, and M5M.

Case group 2: 24 cleaning staff members who received extensive N6M training along the same lines as for case group 1 but addressed HH opportunities for N6M.

Case group 3: 18 cleaning staff members already trained in N6M received enhanced training with on-site teaching to simulate HH practice and behaviour in the work environment. Signs featuring N6M were displayed in the medical waste disposal room, sanitary ware truck, and other primary cleaning areas to serve as a cautionary reminder.

### Observation methods

The observers were a nosocomial infection management specialist and a nursing infection control specialist, and observations were recorded anonymously. The specialists received training before the monitoring and were certified by assessment by non-project group members. The cleaning and disinfection working environment of cleaning staff includes wards, nurses’ stations, treatment rooms, ward corridors, buffer rooms, disposal rooms, medical staff duty rooms, and medical staff restaurants.

During the baseline period, cleaning staff were selected for monitoring for 1 hour of their shift on 2 days; on these days at the end of the intervention month, all participants in the different case groups were each observed for 1 hour; the periods of the observation were consistent with staff working hours. The HH event of cleaning staff include washing hands with water and hand sanitizer and/or using a quick hand sanitizer to clean hands. We took the M5M as the starting point for evaluating all groups since it appears that case groups 2 and 3 did not receive the N6M until the intervention began.

### Outcomes

The main outcomes were as follows: the HHC of the four groups before the intervention; 1, 2, and 3 months after the intervention; compliance at different moments; and time distribution. Compliance measurements were based on actions (X) and opportunities (N), the latter denominator being defined as the moments during healthcare activities when it is necessary to interrupt the hand transmission of microorganisms. Overall compliance was defined as the ratio of the number of actions performed to the number of opportunities.

### Statistical methods

All manual data entries were double-checked for precision. Categorical data are presented as absolute numbers, percentages, and respective 95% confidence intervals (CIs). Continuous variables are presented as mean and standard deviation if normally distributed, or as median and interquartile range if not. The chi-square test or Fisher’s precision probability test was used for comparison between groups. The independent-sample *t*-test was used for comparison between groups. Measurements that did not conform to the normal distribution were described by the median (lower and upper quartiles). The Mann–Whitney rank sum test was used for comparison between groups with a bilateral test level *α* = 0.05. Sample weights were used for all analyses to provide nationally representative estimates with 95% CIs.

Post hoc assessments were conducted according to the different groups and different HH moments. All statistical tests were two-tailed and were considered statistically significant if *p* < 0.05. Statistical analysis was performed using *SPSS* 20.0, and figures were constructed using Python (Version 3.6.6) and *R* software (Version 3.6.1, R Foundation for Statistical Computing).

## Results

### HHC before and after interventions among different groups

The population and sociology data of the four groups were comparable and not significantly different (*p* > 0.05) (Table 1). The control group monitored 106 and 591 HH moments before and after the intervention, respectively. Following the 3-month intervention period, the HHC decreased from the baseline of 30.19% (95% CI, 0.2227–0.3950) to 27.41% (95% CI, 0.2397–0.3114) (Supplementary Figure 1 and Table 2). In case groups 1–3, the number of HH monitoring before and after the intervention was 115 versus 706, 524 versus 645, and 591 versus 519, respectively. The HHCs of case groups 1–3 before and after the intervention were 31.30% versus 42.78%, 23.85% versus 59.22%, and 27.41% versus 83.62%, respectively. The largest increase was noted in case group 3, with an improvement rate of 56.21% (*p* < 0.05) (Supplementary Figure 2 and Table 2).

**Table 1.** Population and sociology data of four groups of cleaning staff

Categories	Control group (*n* = 21)	Case group 1 (*n* = 26)	Case group 2 (*n* = 24)	Case group 3 (*n* = 18)	*P*
Sex					0.404^a^
Male	6 (28.57%)	10 (38.46%)	5 (20.83%)	3 (16.67%)	
Female	15 (71.43%)	16 (61.54%)	19 (79.17%)	15 (83.33%)	
Age (year)	60.14 ± 3.58	60.35 ± 4.53	60.13 ± 4.14	59.94 ± 3.80	0.991^b^
Degree of education					0.940^a^
Primary school	16 (76.19%)	20 (76.92%)	20 (83.33%)	14 (77.78%)	
Middle school	5 (23.81%)	6 (23.08%)	4 (16.67%)	4 (22.22%)	
Working life (year)	2.48 ± 1.12	2.19 ± 0.90	2.25 ± 1.15	2.22 ± 0.81	0.785^b^

a Fisher’s precision probability test.

b One-way ANOVA.

**Table 2.** Comparison of HHC before and after the intervention in different groups

	Before		After 1 month		After 2 months		After 3 months	
Groups	HHC	95% CI	HHC	95% CI	HHC	95% CI	HHC	95% CI
Control group (*n* = 21)	30.19% (32/106)	0.2227–0.3950	35.82% (48/134)	0.2820–0.4423	31.43%^a^ (44/140)	0.2432–0.3953	27.41%^a^ (162/591)	0.2397–0.3114
Case group 1 (*n* = 26)	31.30% (36/115)	0.2355–0.4026	34.15% (56/164)	0.2733–0.4170	38.74%^a^ (74/191)	0.3212–0.4581	42.78%^a^ (302/706)	0.3918–0.4646
Case group 2 (*n* = 24)	23.85% (125/524)	0.2040–0.2768	35.02% (222/634)	0.3141–0.3881	40.19%^a^ (297/739)	0.3641–0.4377	59.22%^a^ ^,^^b^ (382/645)	0.5538–0.6295
Case group 3 (*n* = 18)	27.41% (162/591)	0.2397–0.3114	37.76% (196/519)	0.3369–0.4201	52.21%^a^ (343/657)	0.4839–0.5601	83.62%^a^ ^,^^b^ (434/519)	0.8019–0.8655

a *P* < 0.05 comparison among four groups after the intervention.

b *P* < 0.05 comparison within groups before and after the intervention.

Following the intervention, case group 3 scored the highest HHC at 83.62% (95% CI, 0.8019–0.8655), followed by case group 2 at 59.22% (95% CI, 0.5538–0.6295), case group 1 at 42.78% (95% CI, 0.3918–0.4646), and the control group at 27.41% (95% CI, 0.2397–0.3114) (Supplementary Figure 2 and Table 2).

### HHC of different moments after the intervention

The moments of the highest HHC in case group 2 after the intervention were ‘after doffing personal protective equipment’ at 64.77% (95% CI, 0.5436–0.7394), and the lowest value was ‘before cleaning and disinfection’ at 54.55% (95% CI, 0.4525–0.6354). The HHC was higher in case group 3 than in case group 2 for different moments after the intervention, with the highest being ‘after transferring medical waste from the site’ (90.72% (95% CI, 0.833–0.9504)) compared with the lowest ‘after preparing tools’ (78.33% (95% CI, 0.6638–0.8687)) (Supplementary Figure 3 and Table 3).

**Table 3.** Comparison of HHC after the intervention of different moments between two case groups

	Case group 2 (*n* = 24)	Case group 3 (*n* = 18)		
Group	HHC	HHC	95% CI	*P*
Before cleaning and disinfection	54.55%	80.25%	0.1229–0.3747	<0.001
	(60/110)	(65/81)		
After preparing tools	63.16%	78.33%	0.0039–0.2929	0.056
	(48/76)	(47/60)		
After cleaning and disinfection	55.65%	83.33%	0.1556–0.3840	<0.001
	(69/124)	(80/96)		
After doffing personal protective equipment	64.77%	85.53%	0.0747–0.3286	0.002
	(57/88)	(65/76)		
After transferring medical waste from the site	60.61%	90.72%	0.1926–0.3967	<0.001
	(80/132)	(88/97)		
After environmental sorting	59.31%	81.65%	0.1105–0.3254	<0.001
	(86/145)	(89/109)		

### HH time distribution

**Figure 2.**
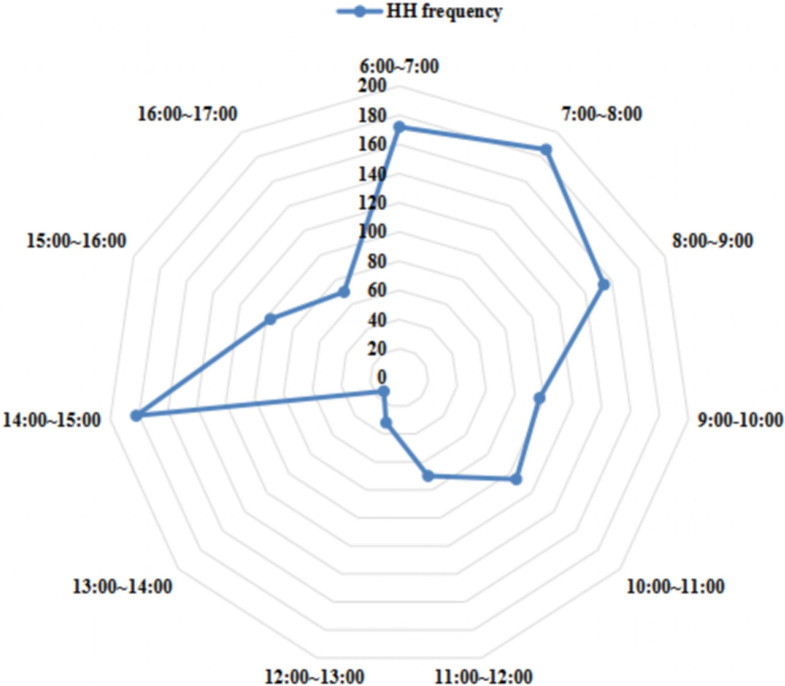
Distribution of hand hygiene frequency of cleaning staff during working time.

The peak hours of the HH activity were 6 AM to 9 AM, and 2 PM to 3 PM, with the monitored activity ranging from 154 to 182 recorded observations (Figure 2).

## Discussion

To the best of our knowledge, our study is the first to analyse the impact of N6M on HHC among hospital cleaning staff, which is an often overlooked but significant demographic in hygiene practice. We observed a significant improvement in HHC for these staff in the N6M intervention group compared with the M5M group. Our findings illustrated that N6M can serve as a technique for HHC monitoring and that a concentrated, relevant training intervention in HH improves practice among cleaning staff.

In our previous study, we found that M5M was most widely used in HHC monitoring and training in medical institutions, but it was not fully applicable to cleaning staff members [16] for whom training in HH moments was lacking and poor compliance monitoring. During the COVID-19 pandemic, the implementation of HH was shown to be particularly important to limit the spread of the virus [17, 18]. Moreover, an earlier survey on knowledge of HH and attitudes showed that hospital cleaning staff members fully understood its importance, but there was an inadequate level of situational training [19], potentially leading to poor HHC practice. The N6M model was formulated according to work situations, and the recognition of hand-touch behaviours of cleaning staff such as ‘before cleaning and disinfection’ and ‘after transferring medical waste from the site’ was incorporated in the model and was more appropriate to the daily work of such staff. Thus, the N6M group comprised a notably larger amount of HH monitoring moments compared with the M5M group, thereby improving the availability and effectiveness of HH monitoring. The sizeable disparity in the number of observations could be attributed to the fact that the opportunities amplified over time and the number of beds for the intervention and occupation were not constant.

Due to the limitations of the medical knowledge of cleaning staff members [20], we adopted a refined and extensive intervention – as proposed by Von Lengerke et al. [14] in our N6M application, which together proved to be superior to the existing M5M interventions. For example, handwashing prompts were posted to create a cultural atmosphere for the cleaning staff to carry out HH in the work environment. As a result, this intervention, as applied in N6M, not only had the best effects on HHC promotion but also stimulated an atmosphere of appreciation of the value of HH practice for cleaning staff.

A further examination was conducted to compare the impact of N6M at different moments in time. This showed that ‘after doffing personal protective equipment’ and ‘after transferring medical waste from the site’ were the two highest HHC moments for these staff members, but compliance with ‘before cleaning and disinfection’ remained low. We speculated that macroscopic contamination such as soiled protective equipment or transfer of medical waste would be sufficiently evident to prompt handwashing by cleaning staff. According to the study of Wen [21] on the HH surveillance of healthcare workers in medical institutions in China, HHC was highest after risk of exposure to body fluids, with doctors and nurses showing almost identical (89.8%) scores.

In addition, the HH timing of cleaning staff had obvious time distribution features which possibly coincided with the heaviest workloads. This implies that monitoring and interventions of infection control practice would be most optimal in these time periods. Moreover, although the nature of cleaning staff work was similar in the four building study areas, differences in patient numbers in various wards would impact on their workloads. For example, the cleaning staff in case groups 2 and 3 were responsible for many patients, and thus a relatively heavier workload often led to increased opportunities for HH. However, 3 months after the intervention, because of the COVID-19 pandemic, the number of patients increased, and they were distributed among the four inpatient buildings. Hence, the number of patients in the control group and case group 1 led to an increase in the workload of cleaning staff and the moments for HH.

Our study had some limitations. First, the number of cleaning staff in the control and case groups was small, which was accounted for by the statistical modelling. Second, the application of the N6M practice was focused primarily on the ward, and not universally applied throughout the buildings. Third, bed occupancy numbers may have impacted negatively on HH opportunities. Lastly, it was a single-centre study in one country. Nevertheless, ancillary and cleaning staff in medical institutions worldwide with different working practices may most likely share similar working characteristics to those studied here, and further adaption of the N6M, or a more locally appropriate similar model, would be worthy of exploration.

In conclusion, the past two decades of advances and perspectives on HH have actively encouraged the adoption of the principle of ‘moments of HH’ in different healthcare populations [4]. In the face of emerging challenges, including but not limited to COVID-19, monkeypox, and drug-resistant bacteria, good HH practice is an effective infection prevention and control strategy, and should be communicated to all grades of clinical and ancillary, which regrettably has for long been lacking. In this context, we consider that our study makes a valuable contribution to the furtherance of the HH practice of staff working on the front line in healthcare facilities.

## Data Availability

The data are available from the corresponding author upon reasonable request.

## Supplementary material

For supplementary material accompanying this paper visit http://doi.org/10.1017/S0950268823000602. click here to view supplementary material
