# Supplementary figures and images for: Exploring the effect of novel six moments on hand hygiene compliance among hospital cleaning staff members: a quasi-experimental study
Source: Epidemiol Infect. 2023 Apr 28;151:e73. doi: 10.1017/S0950268823000602 (PMC10204132; doi:10.1017/S0950268823000602)

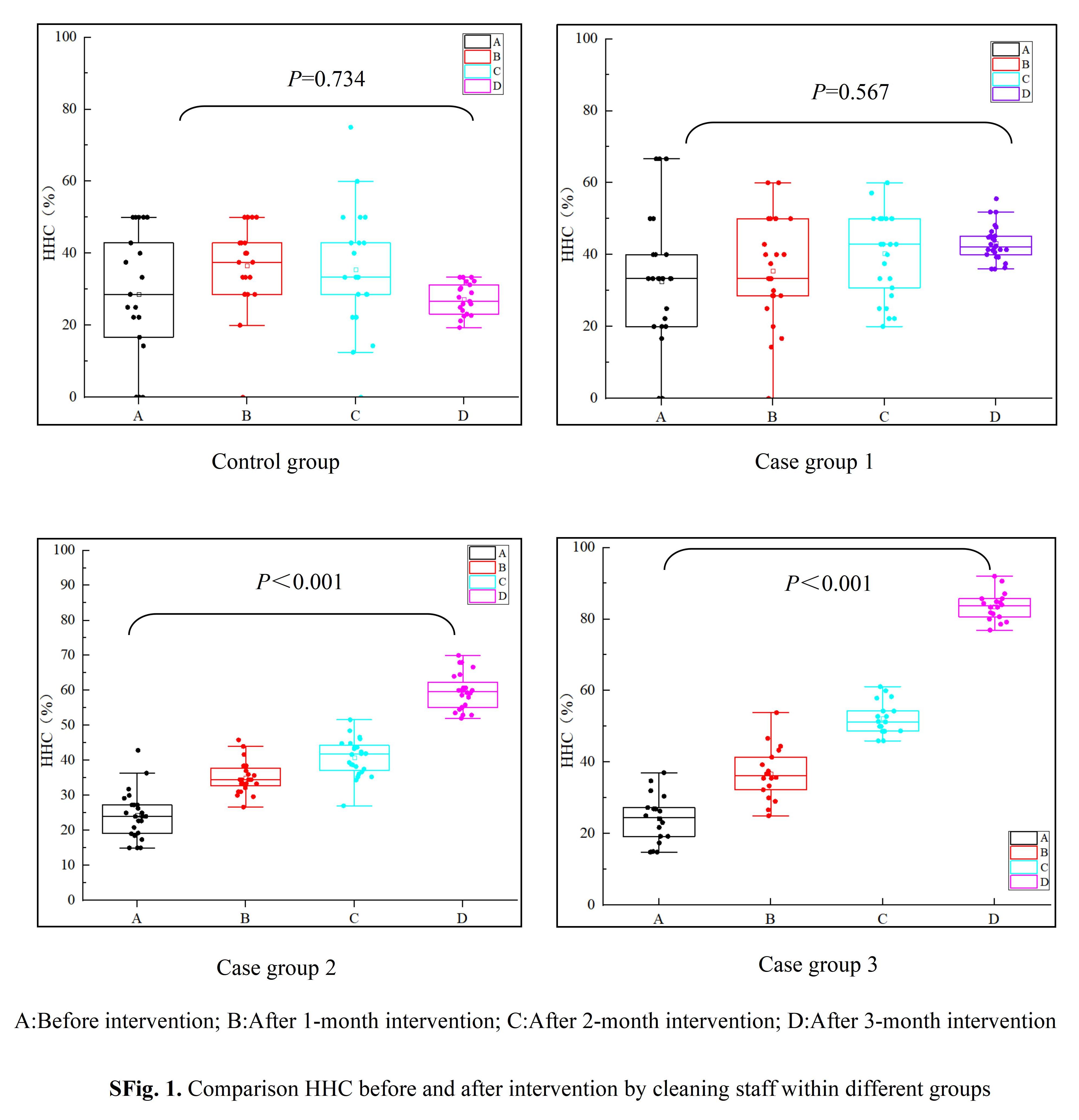

Supplement: Supplementary file 1 [file hygsup.zip › S0950268823000602sup001.jpg]

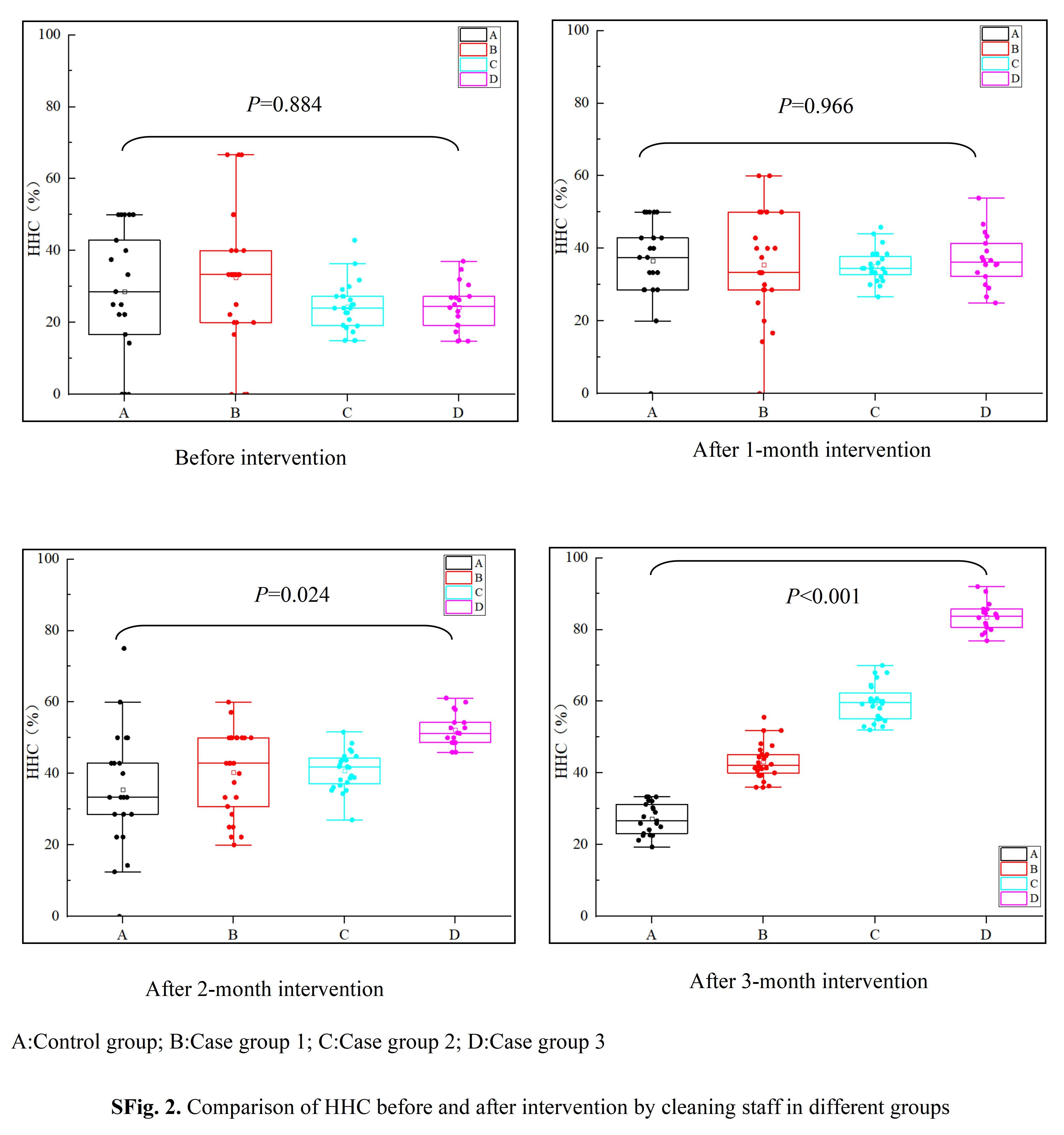

Supplement: Supplementary file 1 [file hygsup.zip › S0950268823000602sup002.jpg]

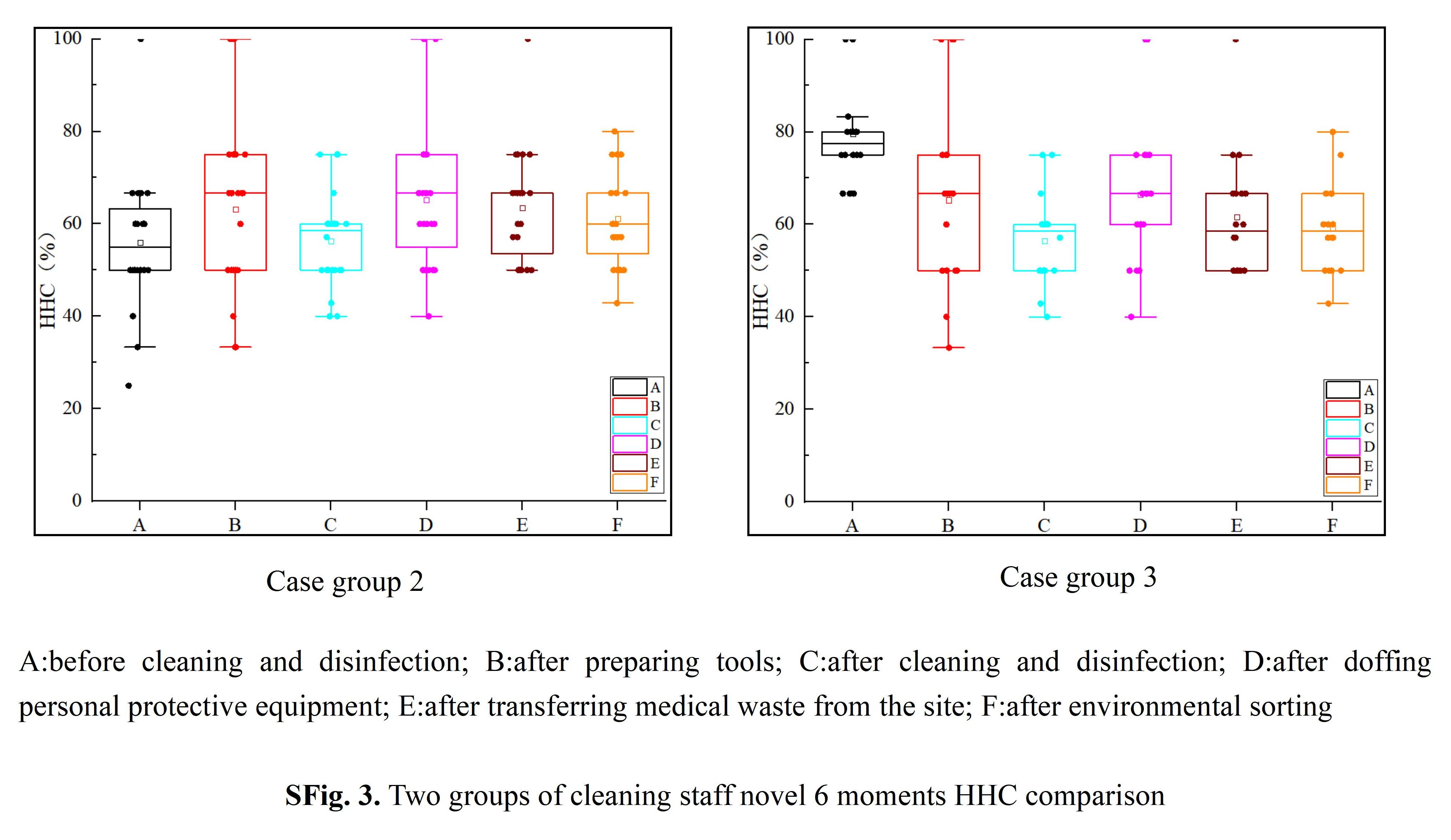

Supplement: Supplementary file 1 [file hygsup.zip › S0950268823000602sup003.jpg]
